# Supplementary material for: Early cellular events of osteomucosal healing in the tooth extraction socket
Source: PLoS One. 2025 Aug 14;20(8):e0328459. doi: 10.1371/journal.pone.0328459 (PMC12352774; doi:10.1371/journal.pone.0328459)
Supplement: Attachment — Submitted filename: [PONE-D-24-55861R1]Kim_2ndRevision_letter (Final).pdf [file pone.0328459.s007.pdf]

May 27, 2025

Dr. Henrique Hadad

Academic Editor,

PLoS ONE

We received your correspondence of April 23rd, 2025, and the comments of the referees concerning our manuscript "**Early cellular events of osteomucosal healing in the tooth extraction socket**" by Kim et al. (PONE-D-24-55861R1) submitted to ***PLoS ONE***. We deeply appreciate the referees' comments. Based on their comments, we have modified the manuscript to correctly address all their concerns. The revisions included in the manuscript are described in our point-by-point responses to the referees' comments detailed below.

Review Comments to the Author

Please use the space provided to explain your answers to the questions above. You may also include additional comments for the author, including concerns about dual publication, research ethics, or publication ethics. (Please upload your review as an attachment if it exceeds 20,000 characters)

Reviewer #2:

The authors have implemented significant revisions to the manuscript, notably enhancing its quality. Among the previous comments provided by this reviewer, the authors have neglected to address one particular issue related to the methods of analysis for the histological and histochemical data. It is recommended that the authors include the detailed methods of analysis for each of the outcome measures evaluated, not limited to microCT.

Although the manuscript is written in formal and intelligible English, a comprehensive revision is necessary to resolve the grammatical inconsistencies that have emerged during the review process.

Our response: Thank you for your suggestion. In response to your suggestion, we have thoroughly revised and expanded the "Materials and Methods" section to include comprehensive and detailed descriptions of the methodologies used for analyzing our histological and histochemical outcomes, including Trichrome and Picrosirius Red staining procedures. Additionally, we have carefully performed a comprehensive grammatical review of the manuscript to address and rectify inconsistencies that arose during the previous revision processes.

Reviewer #6:

The authors set out to investigate the spatial and temporal changes in epithelial, connective and bone tissues, in addition to the presence of osteoclasts during the early stages of osteomucosal healing.

I have seen that this article has already been reviewed by extremely meticulous reviewers, which has certainly made it easier for the current reviewer to make his considerations.

First of all, I would like to point out that the main objective, which was to investigate spatial and temporal changes in epithelial, connective and bone tissues, was not fully achieved. Epithelial tissue was not included in either the methodology or the results. Information on epithelial proliferation and maturation of epithelial layers with keratin formation was not analysed. Immunohistochemistry for epithelial markers such as CKPool and EGFR would have enriched the study. In addition, temporal changes were only made between connective tissue and new bone formation.

Our response: Thank you for raising this important point. While epithelial proliferation and maturation of epithelial layers are important parts of soft tissue healing, we mainly focused on epithelial migration (as determined by wound closure) because it is one of the main clinical criteria of determining whether the tooth extraction sockets are healing or not. Indeed, 1-2 weeks of post-op appointment in a dental office is to examine whether there is any bone exposure remaining. In the normal setting, epithelial proliferation and maturation (e.g., keratinization) follow when epithelial migration occurs successfully and closes the wounded site. Therefore, our focus in investigating the cellular changes in osteomucosal healing is to determine the wound closure as shown in the Fig. 1. Currently, we are working on changes in osteomucosal healing at the molecular level using single cell RNA sequencing (scRNA-seq), preliminary results of which were briefly shared in our previous revised version. We do agree that these molecular analyses will further enrich the study, but we also feel that adding these analyses will not change the conclusion of our study and are beyond the scope of this manuscript. This insight offers meaningful guidance for our future studies.

Materials and Methods
The authors said: "Atraumatic extraction of the maxillary first molars (M1) was carried out". However, rodent maxillary first molars have a variable number of roots and are very thin. This reviewer is curious to know whether root fractures were found. This is important information to clarify for the reader.

Our response: Clinically, atraumatic extraction in human can be performed very predictably and reproducibly when elevators/luxators are effectively used to separate periodontal ligaments (PDL) before using forceps. In mice, we use only explorer, but it is being used as both an elevator/luxator and a forcep. This can be done by allowing enough time to elevate the tooth by engaging at the buccal furcation area of the first molar like an elevator/luxator. When you start "feel" loosening of the tooth from the socket, the tip of the explorer can be rotated to extract the tooth like a forcep. Our laboratory has extensive experience performing mouse tooth extractions, having routinely carried out this procedure for over 10 years. Thus, we are comfortable in the reproducibility and reliability of our extraction methods without root fractures. Although it is extremely rare for us by now, we do seldomly experience root fractures mostly on the M root due to angulation issues, especially when the bone becomes very strong by anti-resorptive agents such as bisphosphonates or anti-RANKL neutralizing antibody. In this study, however, we did not experience root fracture. We have explicitly clarified this point in the revised Materials and Methods section of the manuscript to ensure complete transparency and to avoid any potential confusion for readers as the following (high-lighted below).

> **"µCT Scan**
>
> Areas of interest on the maxillae were subjected to µCT scanning (SkyScan1275, Bruker, Kontich, Belgium) at 60 kVp and 166 µA using a voxel size of 10 μm^3 and a 0.5 mm Aluminum filter with an integration time of 200 ms using a cylindrical tube (FOV/Diameter: 20.48 mm). The Region of Interest (ROI) was defined as the extraction socket, extending from the most coronal aspect of the alveolar bone crest to the apical portion of the socket. Fractured root tips were observed from the µCT images and confirmed no remaining root tips in all analyzed sockets. The ROI was segmented based on grayscale thresholding to differentiate between mineralized and non-mineralized tissues. Two-dimensional slices from each femur were combined using the SkyScan NRecon software to form a three-dimensional reconstruction. The image analysis was performed using the DataViewer and CTAn software."

Immunohistochemical staining

It is important to describe the immunohistochemical evaluation parameters, including the cellular localisation of expression of the two antibodies evaluated. RANK-L is expressed at the cell membrane and in the cytoplasm. Do the authors take this location into account when scoring the samples? What about the anti-osteoprotegerin antibody? It is usually secreted.

Our response: Thank you for this thoughtful comment. In our immunohistochemical

evaluation, we carefully considered the localization patterns of both RANKL and OPG when interpreting the staining results:

For RANKL, positive staining was observed in both the cytoplasm and the cell membrane of stromal and mononuclear cells adjacent to the bone surfaces. Both patterns of subcellular localization were taken into account in our interpretation, as described in the revised Results section. RANKL expression was particularly prominent during the early healing phase (Days 0–4) and became more restricted and less intense by Days 6–7, consistent with its role in initiating osteoclastogenesis during early socket remodeling.

For OPG, consistent with its biological function as a secreted soluble factor, staining appeared primarily as the diffuse extracellular matrix-associated signal, especially surrounding newly formed bone. Rather than scoring individual cell positivity, we interpreted OPG expression based on its distribution pattern and localization within the tissue microenvironment.
Our intent with these IHC experiments was to illustrate the spatial relationship between these two regulators and osteoclast activity over time.

Results
Figure 1(D) Day 3 - I would suggest revising the description of the histological findings. It is possible to see a proliferation of spindle cells at a higher magnification, and this data is important for the healing process.
Figure 1(D) Day 4 - I suggest replacement of the microphotograph from this day, as the histological aspects are worse in the healing process than on day 3. Do the basophilic areas suggest necrosis or the accumulation of polymorphonuclear neutrophils cells?

Figure 1(D) Day 5 - This photomicrograph shows the proliferative pattern of spindle cells. However, there are a lot of wood shavings remaining in the upper field, which could hinder the healing process. Do the authors have another image with no wood shavings to replace it? Figure 1(D) Days 6 and 7 were good.

Our response: Thank you for your detailed feedback on the histological data. To address your suggestions comprehensively, we have retaken and replaced the original images with new micrographs captured under higher resolution and magnification. Specifically:

Day 3: The revised images now clearly illustrate the proliferation of spindle cells under higher magnification. This cellular detail is now explicitly highlighted and described in the revised results section, providing enhanced clarity and stronger support for our conclusions.

Day 4: We have provided a new microphotograph that better represents the healing stage observed on Day 4.

Day 5: We replaced the original photomicrograph with a new image that is free from wood shaving artifacts, allowing clearer visualization of the proliferative spindle cell pattern.

With regard to Figure 2, this reviewer did not see any temporal changes in the alveoli, with Mason's trichrome staining in terms of new bone formation, except on days 6 and 7.
On the other hand, PS Red staining, with and without polarised light, did not contribute to the analysis and actually exacerbated the visualisation of wood shavings within the alveoli on days 4 and 5.
The durability of this set of micrographs should be re-evaluated.

Our response: Thank you for your detailed observation. In response to your comment, we have retaken the Trichrome staining and Picrosirius Red-stained images at higher resolution and magnification to improve visual clarity and minimize interference from wood shavings. The updated images now provide a clearer view of tissue composition and more clearly distinguish temporal changes in the healing sockets.
Additionally, we re-quantified collagen type I and type III deposition using high-magnification images to improve accuracy and enhance the interpretability of the results. We believe these updates significantly improve the quality and reliability of Figure 2 and more effectively illustrate the progression of bone and collagen matrix formation during the healing process.

The authors describe this in the Results section: "Oral mucosal tissue closure following tooth extraction in mice", "By day 1, the blood clot was replaced by a granulation tissue that seemingly consisted of three distinct layers: the residual coagulum layer in the apical portion, the loose provisional matrix layer in the middle of the socket, and the neo-epithelial layers in the coronal portion."

In pathology, granulation tissue is a vascular connective tissue that forms in wounds during the healing process. It's a hallmark of wound healing and is characterised histologically by the presence and proliferation of fibroblasts, macrophages, keratinocytes, endothelial cells, new thin-walled capillaries and inflammatory cell infiltration of the extracellular matrix.
The presence of granulation tissue in a wound means that the inflammatory phase has passed and the proliferative phase has begun. It's important to remember that under normal conditions, wound healing and tissue repair occur in 4 stages: haemostasis, inflammatory stage, proliferative stage (when granulation tissue formation occurs) and remodelling stage.
Therefore, it is not possible to say that the blood clot has been replaced by granulation tissue on day 1.

Our response: Thank you for your important and insightful comment. We agree that the classical definition of granulation tissue refers to a vascularized connective tissue formed

during the proliferative phase of wound healing, following the inflammatory phase. Based on your observation, we recognize that it was premature to refer to the Day 1 wound content as granulation tissue. Accordingly, we have revised the Results section to more accurately describe the Day 1 findings as follows:

> "By Day 1, the extraction socket was filled with a residual blood clot, transitioning toward the formation of a provisional matrix layer consisting of early inflammatory cell infiltration and initial extracellular matrix deposition."

We appreciate the reviewer's clarification and have modified the language throughout the manuscript to ensure alignment with standard pathological definitions of wound healing stages.

Finally, this reviewer would like to know why the teeth in the tomographic and histological images have their roots in the lower part of the image and the crowns in the upper part, simulating teeth from the lower arch. Have the upper/maxillary first molars not been extracted? If so, the roots should be in the upper part of the images.

Our response: Thank you for this helpful observation. We confirm that all images in the study represent maxillary first molars. To improve consistency and ease of interpretation, we chose to orient the tomographic and histological images with the crown at the top and the roots at the bottom across all time points. In the field of medicine where skin wounds and healing are being described, basal cell layers are typically placed in the bottom while keratinized layers are placed on the top regardless of the human anatomical parts (e.g., sole foot). Similarly, in dentistry, this orientation of epithelial tissues and tooth crown being on the top while bone tissues and roots being in the bottom mirrors the layout commonly used in histological sections and allows for easier side-by-side comparisons between modalities.
To prevent confusion, we have revised the figure legends and relevant methods text to explicitly state that these images are from the maxillary first molars and that the orientation was standardized for visual clarity.

Please correct these words:

Such difference is largely owing to multiple layers of in-beween (in-between) anatomical structures such as muscle, fat, or fascia that separates soft and hard tissues.
…..analysis revealed that both collagen type I (yellow/organge (orange)/red) and III (green) followed a similar……

Our response: Thank you for carefully reviewing our manuscript and highlighting these typographical errors. We have now corrected these mistakes. Additionally, we have carefully reviewed the entire manuscript again to ensure no further typographical errors remain.

Reviewer #7:

This revised manuscript investigates the early healing process in tooth extraction sockets. As the authors note, morphological analyses provide valuable insights into post-extraction healing. The selected methodologies are generally appropriate; however, the resolution and depth of the observations are limited, and as such, they fall short of offering substantial mechanistic insight into the healing process. Detailed comments are provided below.

1. Oral and Mucosal Tissue Closure Following Tooth Extraction in Mice (Figure 1)
The authors describe bone resorption mediated by osteoclasts; however, based on the current image resolution, osteoclasts cannot be clearly identified.

Figure 1D: The image size and resolution are insufficient to support the conclusions drawn in the results. Since these images are foundational to the study's claims, they should be presented in higher resolution and at larger sizes to improve clarity and impact.

Our response: Thank you for pointing out the importance of image resolution and clarity in supporting our conclusions. In response, we have carefully revised Figure 1D by providing H&E-stained images at higher resolution and magnification. These revised images clearly differentiate tissue structures and allow better visualization of the cellular details, including the precise localization and identification of osteoclasts involved in bone resorption. We believe these improvements significantly enhance the clarity, reliability, and interpretability of our histological data.

2. Collagen Deposition in the Tooth Extraction Sockets (Figure 2)

The rationale for this analysis, particularly the distinction between Type I and Type III collagen, needs to be explicitly stated. Additionally, a detailed explanation of the spatial distribution of these collagen types within the healing socket should be provided.
While Picrosirius Red staining under polarized light is commonly used to assess collagen types, it is not definitive. Therefore, confirmation via immunohistochemistry is necessary.

The image quality, especially under polarized microscopy, is not sufficient to clearly distinguish collagen types or their distribution. Larger, higher-resolution images are required to support the data.

Our response: We appreciate the reviewer's thoughtful comments regarding collagen identification methods. While the immunohistochemical (IHC) staining may be necessary to confirm the finding for Collagen Type I and III, Picrosirius Red (PSR) staining combined with polarized light microscopy is a well-established and widely accepted technique for

differentiating collagen types based on their birefringent properties [1-5]. Numerous studies have demonstrated a strong correlation between PSR staining and immunohistochemical (IHC) approaches in identifying collagen types I and III. Furthermore, we felt that adding IHC for Collagen Type I and Type III will not make any changes in our conclusion in the study where collagen formation proceeds bone formation. Nonetheless, to address and elaborate on the reviewer's concern, we have retaken PSR-stained images both with and without polarized light at higher resolution and magnification to enhance clarity (Please refer to our response to Reviewer #6). These updated images allow improved visualization of collagen fiber organization and more distinct differentiation between collagen types. Furthermore, we re-quantified collagen type I and III using these high-magnification images to ensure a more accurate and representative analysis of collagen deposition over time.

While IHC provides molecular specificity, the PSR-polarization technique remains a robust and reproducible method for histological assessment of collagen types in situ. Given the observational nature and scope of our current study, we believe that the enhanced image quality and re-analysis sufficiently support our conclusions regarding collagen organization during early socket healing.

3. Woven Bone Formation in the Tooth Extraction Sockets (Figure 3) The purpose of this analysis should be clearly stated. In the discussion, the authors note the continuity between woven and pre-existing bone, and the woven bone is formed independently within the healing connective tissue. It is an important observation, and thus should be explicitly addressed here. Micro-CT 3D reconstruction could clarify whether distinct bone formation, such as appositional growth on existing bone surfaces versus de novo intramembranous ossification, is occurring. Additionally, further exploring these distinct processes associated with molecular regulation would greatly enhance the depth of the study.

In Panel A, the distobuccal (DB) socket appears to exhibit earlier woven bone formation compared to the mesial (M) socket. The authors should acknowledge this and analyze whether healing differs significantly between the two sites.

Our response: We really appreciate the reviewer for acknowledging this important observation. We, too, believe that this is a very important observation to differentiate appositional bone growth from the existing bone surfaces vs. de novo intramembranous ossification, which was already experimentally demonstrated from our data in the Fig. 3B, top panel. As such, we have added the following sentence in the Result section.

> "Interestingly, 3D reconstructions of the woven bone formation within the socket showed that some of these mineralized tissues are not continuous with the existing bone surface

from the socket, suggesting that they formed by *de novo* intramembranous ossification. (Fig. 3B and Fig. 3B top panel)."

The purpose of this analysis was to examine the temporal and spatial progression of woven bone formation within the extraction sockets using longitudinal μCT imaging and 3D reconstruction. In particular, we aimed to assess when and where de novo intramembranous ossification occurs during early socket healing.

As noted, in Panel A, woven bone appears to form earlier in the distobuccal (DB) socket compared to the mesial (M) socket based on longitudinal μCT images (Fig. 3A). However, upon further analysis, we believe this is primarily due to the smaller anatomical volume of the DB root, which makes early bone deposition more visually prominent in that region. To address this point, we performed separate quantitative analyses for the DB and M regions (Fig. 3C–D). These analyses confirmed a significant increase in woven bone volume by Day 7 in both regions. However, no statistically significant difference in the rate or volume of bone formation was found between the DB and M sockets.
The Results section has been revised accordingly.

4. Osteoclast Formation in the Tooth Extraction Sockets (Figure 4) To better differentiate between osteoclasts associated with newly formed versus pre-existing bone, magnified insets of these respective areas should be provided. This would allow clearer visualization of osteoclast localization relative to bone context.

Our response: Thank you for this valuable suggestion. In response to your recommendation, we have updated Figure 4 to include images captured at higher magnification. These enhanced views clearly distinguish osteoclasts located on newly formed bone from those associated with pre-existing alveolar bone. To further improve clarity, we have added dot arrows to explicitly highlight osteoclasts on the newly formed bone surfaces. In addition, the Results and Discussion have been revised accordingly. We believe these enhancements substantially improve visualization and interpretation, enabling readers to more clearly appreciate the spatial context of osteoclast activity within the extraction socket.

"Result

Osteoclast formation in the tooth-extraction sockets in mice

To evaluate the dynamics of osteoclast recruitment during early wound healing, TRAP staining was performed on maxillary extraction sockets. No TRAP-positive osteoclasts were observed immediately after extraction (Day 0). By Day 2, a small number of multinucleated TRAP-positive cells appeared, localized primarily along the edges of pre-existing alveolar bone surfaces (Fig. 4A). The number of TRAP+ cells increased significantly between Days 3 and 5, peaking on Day 5, and remained elevated through Day 7 (Fig. 4B). Interestingly, osteoclasts were observed

exclusively along the edges of pre-existing alveolar bone during the early time points (Days 2–4). However, beginning on Day 5, TRAP-positive cells became detectable on newly formed bone trabeculae when examined under higher magnification (Fig. 4A, dashed arrow). These findings suggest that osteoclasts also participate in the early remodeling of de novo bone, in addition to their role in resorbing pre-existing bone margins.

Discussion

It is worth noting that TRAP+ osteoclasts were initially localized predominantly on the surface of pre-existing alveolar bone during the early healing phase (Days 2–4). However, by Day 5, osteoclasts were also observed on newly formed woven bone trabeculae, as confirmed under higher magnification (Fig. 4A). This spatial shift suggests a transition in osteoclast function—from remodeling existing bone to engaging in the early remodeling of de novo bone structures within the healing socket."

5. Expression of RANKL and OPG in the Tooth Extraction Sockets (Figure 5) Since RANKL is produced not only by osteoblasts but also by lymphocytes and stromal cells, the authors should carefully delineate the association of RANKL-positive staining with specific cell types and tissue structures.

To confirm the relationship between RANKL expression and osteoclast activity, double staining for RANKL and TRAP is recommended.

Our response: Thank you for raising this insightful comment. RANKL is indeed expressed by multiple cells, including osteoblasts, stromal cells, and lymphocytes. It is important to identify which cells mainly express RANKL during the early stages of osteomucosal healing, as it may help regulating formation and functions of osteoclasts. This is an important question that is under active investigation using scRNA-seq, results of which were briefly discussed in the previous revised letter, and we feel that this is beyond the of this study without altering the conclusion of this study.

Regarding the recommendation for double staining of RANKL and TRAP, we respectfully point out that expression patterns of RANKL and TRAP are different: osteoclasts, characterized by TRAP positivity, are not typically considered primary sources of RANKL. Rather, osteoclast formation and activation are regulated through RANKL produced by osteoblast-lineage cells, osteocytes, and stromal cells. As such, RANKL and TRAP stainings do not co-localize. Thus, performing double staining for RANKL and TRAP would primarily highlight the spatial proximity of osteoclasts to RANKL-producing cells but would not necessarily provide additional mechanistic insights beyond our current findings.

6. Overall Comments

The current dataset does not provide sufficient depth to fully elucidate the regulatory mechanisms underlying socket healing. Given the breadth of existing literature on bone wound healing, the authors should contextualize their findings with key molecular events known to occur during skeletal repair.

Moreover, tooth extraction results in disruption and torsion of collagen fibers from the periodontal ligament (PDL) that insert into the alveolar bone, likely causing structural changes to the bone surface. The mechanical load during tooth extraction may also affect osteocytes adjacent to the bone surface. Beyond the observed difference in the osteoclast distribution, the authors need to demonstrate the possible causes that could influence osteoclast recruitment and socket bone remodeling during the healing process. Investigating these structural and cellular dynamics could offer important insights into the interplay between immune responses, stromal cells, and osteoclastogenesis in the healing socket.

Our response: We greatly appreciate the reviewer's thoughtful feedback. As the review correctly pointed out, structural changes after tooth extraction are likely due to disruption and torsion of PDL and changes in osteocytes next to the bone surface after mechanical trauma from extraction. Add to these changes, we also have to consider infiltration of inflammatory cells (including monocytes/macrophages which are pre-cursor cells of osteoclasts) as well as the presence of different strains of oral bacteria. Furthermore, genetic make-up of the hosts that are required for proper osteomucosal healing is equally important to identify molecular events during the osteomucosal healing. All of these topics, which are so important to fully elucidate in-depth regulatory mechanisms, are still underexplored areas which require a dedicated manuscript for each topic. Our study is meant to describe early cellular events in the osteomucosal healing, which will serve as a foundational observational work to further broaden mechanistic investigations of every topic listed above.

That being said, we fully agree with the reviewer that understanding the molecular and mechanistic underpinnings of socket healing is essential for gaining a more comprehensive view of the biological processes involved. Based on the findings from this study, we have already initiated follow-up research that will leverage scRNA-seq to investigate the cellular and molecular crosstalk among bone, soft tissue, and immune cells during the healing process. This next phase of work is specifically designed to address the mechanistic questions raised by the reviewer, including the interplay between immune responses, stromal cells, and osteoclastogenesis within the socket microenvironment.

In the Discussion Section, we have correctly pointed out that the limitations of our study as follows.

“Lastly, our study focused on cellular changes during osteomucosal healing. Behaviors of the cells involved in the wound healing processes are primarily governed by molecular cues from their microenvironments. As such, additional molecular studies should be followed to support the findings observed in this study.”

References

1. Coelho PGB, Souza MV, Conceicao LG, Viloria MIV, Bedoya SAO. Evaluation of dermal collagen stained with picrosirius red and examined under polarized light microscopy. An Bras Dermatol. 2018;93(3):415-8. doi: 10.1590/abd1806-4841.20187544. PubMed PMID: 29924246; PubMed Central PMCID: PMCPMC6001092.
2. Drifka CR, Loeffler AG, Mathewson K, Mehta G, Keikhosravi A, Liu Y, et al. Comparison of Picrosirius Red Staining With Second Harmonic Generation Imaging for the Quantification of Clinically Relevant Collagen Fiber Features in Histopathology Samples. J Histochem Cytochem. 2016;64(9):519-29. Epub 20160722. doi: 10.1369/0022155416659249. PubMed PMID: 27449741; PubMed Central PMCID: PMCPMC5006137.
3. Zhang D, Fu Q, Fu H, Zeng J, Jia L, Chen M. 3D-bioprinted human lipoaspirate-derived cell-laden skin constructs for healing of full-thickness skin defects. Int J Bioprint. 2023;9(4):718. Epub 20230323. doi: 10.18063/ijb.718. PubMed PMID: 37323499; PubMed Central PMCID: PMCPMC10261198.
4. Sarojini H, Billeter AT, Eichenberger S, Druen D, Barnett R, Gardner SA, et al. Rapid tissue regeneration induced by intracellular ATP delivery-A preliminary mechanistic study. PLoS One. 2017;12(4):e0174899. Epub 20170405. doi: 10.1371/journal.pone.0174899. PubMed PMID: 28380006; PubMed Central PMCID: PMCPMC5381896.
5. Seifert AW, Monaghan JR, Voss SR, Maden M. Skin regeneration in adult axolotls: a blueprint for scar-free healing in vertebrates. PLoS One. 2012;7(4):e32875. Epub 20120402. doi: 10.1371/journal.pone.0032875. PubMed PMID: 22485136; PubMed Central PMCID: PMCPMC3317654.
