# Supplementary material for: Early cellular events of osteomucosal healing in the tooth extraction socket
Source: PLoS One. 2025 Aug 14;20(8):e0328459. doi: 10.1371/journal.pone.0328459 (PMC12352774; doi:10.1371/journal.pone.0328459)
Supplement: Attachment — Submitted filename: Responses to reviewers.docx [file pone.0328459.s006.docx]

Responses to Reviewers

We deeply appreciate the referees’ comments. We prepared our point-by-point responses (below in blue) and modified the manuscript to correctly address all their concerns. Please see below for our answers.

------------------------------------------------------------------------------------------------------------------

**REVIEWER #1:**

**This is a nice study with an easy-to-read flow, that gathers attention from the reader. Minor questions and suggestions should be assessed by the authors:**

***1.1) When the authors cite MRONJ in the discussion panel, it should be discussed with another statement " The early presence of osteoclasts allows for bone resorption at the existing bone surface and triggers osteoclast-osteoblast coupling to stimulate new bone formation".***

Our response: Thank you for your suggestion. We have included a paragraph dedicating for the clinical implications of our study. In there, we discussed MRONJ more in depth as the following.

***1.2) The authors should discuss the timing of socket cicatrization. It is interesting and not well addressed in the literature how soft tissue and hard tissue heal at different paces but a similar finish line.***

Our response: Thank you for your insightful comment. We now have included a paragraph in the Discussion Section regarding cicatrization, as to why scar formation after healing in the oral cavity is less based on our study. The following paragraph is added.

***1.3) This is a great study, with interesting results. Image 06 does not represent this study, but a grade school paperwork. The authors should present a professional Graphic for*** *Image 06.*

Our response: Thank you for your constructive feedback. We have revised Figure 6 to ensure a professional and polished presentation.

**REVIEWER #2:**

*The manuscript is focused in evaluating epithelial regeneration, connective tissue proliferation and bone formation over the first week following tooth extraction in mice. The authors claim this is unprecedented data, however, the cascade of events occurring during socket healing is well documented. Maybe, their design (consecutive euthanasia over the week) might have added information, but the authors failed to make clear which gap was filled by their data. Besides this limitation that should be addressed, some other major changes need to be done prior to re-consideration for publication, once the authors succeed in*

***2.1) Use the background to state the singularity of osteomucosal healing and exactly which information is been added by your research. “Early events” is vague.***

Our response: Thank you for your comments. We added additional background information, and we emphasized the importance of this study by stating that this study examined spatial and temporal changes in soft and hard tissues. We have revised the abstract by adding the following statement in the beginning.

“Healing in the oral cavity after dentoalveolar trauma such as tooth extraction is a unique process that involves osteomucosal healing - healing of soft and hard tissues at the same time - through hemostasis/coagulation, inflammation, proliferation, and modeling/remodeling stages. Although healing process of soft or hard tissues is well-documented previously independent to each other, the progression of simultaneous healing processes of both during the early time points remain unclear. In this study, we investigated spatial and temporal patterns of epithelial closure, collagen deposition, osteoclast formation, and woven bone development during the early stages of osteomucosal healing.”

***2.2) The rationale is currently based in distinct healing across tissues. Instead, the introduction should focus on the unknown events in osteomucosal healing and plausible crosstalk between the events occurring within the socket.***

Our response: Thank you for this insightful suggestion. Based on the Reviewer’s suggestion, we have revised the Introduction as following.

“Previous studies on osteomucosal healing patterns have been carried out in both humans and animals, mainly focusing on the weeks and months following an injury [6-8]. Although these studies provided valuable insights into tissue remodeling and bone regeneration, critical early events including spatial and temporal coordination of soft and hard tissue healing within the first week after extraction have been underexplored. In this study, we aim to address this knowledge gap by examining the early stages of the osteomucosal healing process within the first week following tooth extraction in mice. Specifically, we examine the kinetics of epithelial wound closure, connective tissue formation, and new bone formation, as well as monitor the formation of osteoclasts and the expression of OPG and RANKL at the tooth extraction sites.”

***2.3). Did you perform sample size calculation? Based on most literature of socket healing in mice, N=3 seems not sufficient to assure relevance. You mention that both right and left first molars were extracted, which is different from the abstract. Which one is correct?***

Our response: Thank you for asking this clarification. In our study, N=3 refers to the number of mice per group, with both the maxillary right and left first molars extracted from each mouse, resulting in **6 total extraction sockets per group at each time point.** To ensure clarity, we have revised the Materials and Methods section to explicitly state that each group consisted of 6 sockets (2 sockets per mouse × 3 mice) as the following.

“Three mice were sacrificed every day for 7 days, resulting in a total of 6 extraction sockets per group per time point (2 sockets per mouse × 3 mice per time point).”

***2.4) Describe how you defined the region of interest and the method of analysis for the microCT. Mason’s Trichrome was only mentioned in the results and the information is lacking in the M&M. The method of analysis for all outcome parameters was neglected or poorly described. An extensive revision on that is necessary.***

Our response: Thank you for pointing out the need for more detailed descriptions of our methodologies. We have revised the Materials and Methods section as the following.

“The **Region of Interest (ROI)** was defined as the extraction socket, extending from the most coronal aspect of the alveolar bone crest to the apical portion of the socket. The ROI was segmented based on grayscale thresholding to differentiate between mineralized and non-mineralized tissues.”

***2.5) The clinical analysis should focus on wound closure only, instead of describing tissues. Epithelium can be appreciated in the histological slides. The word “matured” should be changed to proper histologic terminology. The figures are shown in poor quality, hindering a proper appreciation of their representativeness. Additionally, they are presented in low magnification, which compromises a proper visualization of the tissue events described in the text. The IHC is also shown in low magnification. I understand that the labeling is not confined to the cytosolic compartment since these proteins exert their function when secreted in the extracellular matrix. However, higher magnification images are necessary to identify what is antigen-antibody labeling and what is background labeling.***

Our response: Thank you for your detailed feedback. We have made significant revisions to address these points:

🡪 Focus on Wound Closure: In the revised Results section, we have focused the analysis on wound closure dynamics and removed unnecessary descriptions of the surrounding tissues.

🡪 Terminology Update: The term "matured" has been replaced with proper histological terminology. Specifically, we now use terms like "fully developed connective tissue" to align with standard histological language.

🡪 Improved Figure Quality: The current magnification was deliberately chosen to capture the entire socket area and provide an overview of the morphological changes occurring during the healing process. We recognize that this broader view may lack the detailed resolution needed for certain features, so we have added additional higher-magnification images of key areas to provide greater clarity.

***2.6) Overall, the discussion is missing comprehensive understanding of the data shown by the authors. Also, the authors could explore the sequential (and overlapping) events trying to establish a spatial relevance of uneventful osteomucosal healing.***

Our response: Thank you for your valuable feedback. In the Discussion Section, we have revised accordingly so that we discuss about the spatial and temporal events occurred during osteomucosal healing. In particular, we discussed about epithelial closure first (2^nd^ paragraph), then collagen formation (2^nd^ paragraph) followed by bone formation (3^rd^ paragraph). After that, we focused on discussion about osteoclasts (4^th^ and 5^th^ paragraphs), explanation of which is foundational to our further discussion about 3 clinical implications – dry socket, MRONJ, and scarless formation.

***2.7) A proper conclusion based on the results should be drawn.***

Our response: Thank you for your constructive feedback. We have included the Conclusion paragraph at the end of the Discussion Section.

**REVIWER #3:**

The proposed paper details the first week of alveolar repair in daily analyses. A very interesting study that will certainly support studies regarding interferences in the natural course. The objectives are clear and the methodology is consolidated in the literature in relation to alveolar repair.
It precisely details the role of osteoclasts in the early stages after tooth extraction, which is well explored in the discussion as possible indicators for studies of the mechanisms of MRONJ.
Based on the analysis of the study, I suggest some small changes:

***3.1) There is a tendency in the results section to repeat some points from the methods, for example:
- “To investigate the kinetics of collagen formation and deposition following tooth extraction, we stained the sections with Trichrome, a maker of collagens in general…”
I suggest that these points be part of the method only and not repeated in the results section.***

Our response: Thank you for your feedback. We have revised the Results section to reflect the Reviewer’s suggestion. Here is one example.

Previous version:

“To investigate the kinetics of collagen formation and deposition following tooth extraction, we stained the sections with Trichrome, a maker of collagens in general, and found that collagen deposition occurs as early as day 2 (Fig. 2A). Picro-Sirius Red staining revealed that collagen type I (yellow/organge/red) and III (green) followed a similar pattern; they started occurring as early as day 2 post-extraction and exponentially increased by day 7 (Fig. 2B and 2C).”

Revised version:

“Collagen deposition was observed as early as day 2 post-extraction (Fig. 2A, top row). Further analysis revealed that both collagen type I (yellow/organge/red) and III (green) followed a similar temporal pattern, appearing on day 2 and increasing exponentially by day 7 (Fig 2A, middle and bottom rows; Fig. 2B and 2C). Statistical analyses across time points indicated a significant increase in collagen deposition between day 6 and day 7 (p < 0.05), indicating rapid extracellular matrix production during this period.”

***2. Regarding Figure 6: although it seems obvious that the markings on the horizontal axis refer to the 7 days studied, I suggest that the numbers of the days to which the markings refer be written. In the same way, markings be added to the vertical axis.***

Our response: Thank you for the comments. We modified the graph as your suggestion.

***3. In the caption of Figure 1, I suggest removing "N = 6 tooth extraction sites with 3 mice per group.", This refers to the method and is already very clear in the text.***

Our response: Thank you. We deleted this statement. Also, please refer to Reviwer #2 Q3 for further clarification.

**Reviewer #4:**

*Kim et al. presented an original study investigating early cellular and molecular events in osteomucosal healing in a mouse model. This study provides a new understanding of collagen deposition, osteoclast activity, and remodeling. The results have clear clinical implications for MRONJ and dry sockets using advanced imaging and staining techniques. Overall, the manuscript provides valuable contributions to the understanding of osteomucosal healing. Its clarity and impact will be further enhanced by addressing the following suggestions.*

***4.1) Abstract: While the abstract is informative, it lacks brief details on the materials and methods used. Including this information would improve clarity and help readers understand how the results were obtained.***

Our response: Thank you for this suggestion. We have revised the abstract to include brief details of the materials and methods used in our study.
 ***4.2) Although the figures and results are robust, the discussion could be more explicit in linking the results to potential future studies on clinical implications, such as MRONJ or dry socket.***

Our response: We have revised the discussion to include explicit connections between our findings and potential clinical implications as below.

“These observations have several important clinical implications. First, tooth extraction often leads to alveolar osteitis (AO) also known as “dry socket.” AO commonly occurs when the blood clots become partially or totally dislodged from the socket, and healing of the dry socket may last several weeks [27]. Based on our study, it is possible that the lodged blood clots may remove an opportunity to induce *de novo* bone formation in an osteoclast-independent manner, leaving the bone formation mode only from the existing bone surfaces that is osteoclast-dependent, a process of which can be slow and further delays the proper osteomucosal wound healing process. Second, in patients taking anti-resorptive agents such as bisphosphonates or denosumab, the osteoclast-osteoblast coupling mechanism is compromised due to inhibition of osteoclast formation and/or functions [28]. As such, bone formation in the socket is primarily dependent on *de novo* bone formation in the blood clots. In these patients, if the blood clots become dislodged after tooth extraction, or if bleeding does not get induced, it may lead to medication-related osteonecrosis of the jaw (MRONJ). Lastly, wound healing in the oral cavity is known for expedited healing with minimal cicatrization, and this scar formation is primarily mediated by dysregulated production of collagens by fibroblasts. Recent studies suggest that microanatomic differences of fibroblasts lead to scar formation; fibroblasts residing in the deep layers are more prone to cause scar formation when compared to the those in the superficial layers [2]. As such, it is possible to speculate that deeper fibroblasts may contribute more to bone formation, rather than scar formation, that leads scarless healing unique to the oral cavity. Further studies await confirmations of these interpretations.”

***4.3) The narrative would be streamlined, and readability would be improved by reducing redundancy in the introduction and discussion.***

Our response: We have streamlined and extensively revised the introduction and the discussion by removing redundant statements and reorganizing the text for better readability.

***4.4) This study did not use additional molecular methods, such as qRT-PCR for gene expression analysis, that could have deepened the findings.***

Our response: Thank you for this insightful comment. We agree to the reviewer, and we have changed the title to limit to cellular events only. While our current study focused on histological and immunohistochemical characterization of early osteomucosal healing, we recognize the importance of molecular methods to further elucidate the underlying mechanisms. To this end, we have already established a follow-up study using single-cell RNA sequencing (scRNA-seq) to investigate gene expression at the cellular level at 1 week after extraction (Revision Fig. 1). This approach will allow us to identify specific cell populations, and their gene expression profiles, providing a more detailed understanding of the molecular pathways driving osteomucosal healing. We add this figure for the reviewer purposes only.

*4.5) Potential limitations, such as differences in healing mechanisms between mice and humans, should be clarified. This would better contextualize the findings.*

Our response: We acknowledge the importance of addressing species-specific differences. We have added a dedicated section discussing the limitations of extrapolating findings from murine models to humans as the following.

“This study provides important insights about the cellular events during the early stages of osteomucosal healing; however, it has several limitations. First, this study is pilot in nature with 6 extraction sites per group (n = 3 mice). Although our study conferred meaningful results with statistically significant differences, more robust analysis with increased numbers of mice and extraction sockets may need to generalize the findings. Second, it is worth noting that there are differences in healing rates, tissue composition, and immune responses between rodents and humans. Although there is no universal “conversion rate” between rodent and human pathophysiological processes [30], healing in rodents occurs relatively faster than that in humans [31]. Therefore, specie differences must be considered when extrapolating these results to clinical scenarios. Lastly, our study focused on cellular changes during osteomucosal healing. Behaviors of the cells involved in the wound healing processes are primarily governed by molecular cues from their microenvironments. As such, additional molecular studies should be followed to support the findings observed in this study.”

**Reviewer #5:** I was pleased to review this manuscript. The initial stages of healing in fresh sockets remain highly important and intriguing. However, the paper lacks adequate support from the existing literature, despite the availability of a substantial works on the topic that could better support the study.

I recommend major review based on the following comments:

*5.1) Abstract: I suggest restructuring the abstract, as the study design is not well described, nor are the results and conclusion clearly presented. Additionally, consider adding keywords to improve the visibility of your study in database searches.*

Our response: Thank you for your comments. We have revised the abstract to address the reviewer’s comments and improve clarity. Additionally, we included the keywords in the title page (first page).

*5.2) Introduction: The introduction lacks sufficient references to support the context and rationale of the study. While the aim is stated, there is no clear hypothesis. What did the authors expect to find or address within this gap?*

Our response: We have revised the introduction to include more background information and supporting literature. Furthermore, we have clarified the hypothesis of the study as following.

“Specifically, we hypothesize that there are spatial and temporal changes in soft and hard tissues that are interdependent to each other during the osteomucosal healing.”

*5.3) Methods: Methodologically, this is essentially a result based on histomorphometric parameters, as you did not explore deeply into gene and protein expression in the samples deeply. It is important to strengthen what is presented. Alternatively, you could change the approach to frame it as a pilot study investigating the initial events of alveolar remodeling.*

Our response: Thank you for highlighting this important point. We agree that the study primarily relies on histomorphometric parameters and does not include gene or protein expression analyses. As such, we framed it as a pilot study in the abstract.

Another reviewer also raised a similar concern, and we have acknowledged this as a limitation in the discussion. To address this, we have already begun exploring a follow-up study utilizing single-cell RNA sequencing (scRNA-seq) to investigate gene expression profiles at the cellular level within the wound socket. This approach will enable us to identify specific transcriptional pathways involved in epithelial closure, collagen deposition, and bone remodeling (Please refer to the answer #4.4). We have included this information for the review purposes only.

*5.4) Did the authors perform a sample size calculation? Considering that the number of animals per time point is very low, with only three animals per period, this would not provide sufficient statistical confidence, even with the use of a split-mouth design. doi: 10.4103/0976-500X.119726*

Our response: Thank you for raising this important concern. The sample size was determined based on ethical considerations and standard practices in the animal experimental model. Technically speaking, this study is not a split-mouth design; we extracted both right and left maxillary molars and included them as an independent sample. This is a reasonable study design while minimizing animal numbers (recommended by the ARC committee) because they are all wildtype same strain of mice, and subjected to normal courses osteomucosal healing without any anticipated difference between right and left sides. While this approach maximized the data obtained per animal, we do recognize its limitations in achieving stronger statistical confidence. Therefore, as the reviewer #5 suggested in 5.3, we have noted it as a pilot study.

*5.5) "Tooth-extraction mouse model" : What instrument was used for the atraumatic extraction? If your article is published, it must be reproducible, allowing other researchers to replicate the study. Additionally, others may draw inspiration from your work for future studies.*

Our response: Thank you for highlighting the importance of specifying the instruments used in our tooth-extraction model. We have revised the Methods section to include a precise description of the instruments used for the atraumatic extraction procedure as the following.

“Atraumatic extraction of the maxillary first molars (M1) was carried out using #5 double-end Explorer (Hu-Friedy, Chicago, IL) as described previously [15, 16]. Briefly, the explorer was carefully placed between the mesial and distal buccal roots, and gentle buccolingual motions were applied to loosen the tooth. Once sufficient mobility was achieved, the tooth was lifted out of the socket with minimal disruption to the surrounding bone and soft tissues. The extraction sockets were then gently irrigated with a sterile cotton ball to remove excess bleeding, and the animals were monitored postoperatively for signs of distress or complications.”

*5.6) μCT Scan: The parameters evaluated are not described, nor are they presented in the results. I suggest using parameters from the literature, such as those described in the study by Viera (2015), as cited by the authors. Histochemical Staining: Improve the description, there is no cited eosin staining with hematoxilin.*

Our response: Thank you for your valuable suggestions. We have addressed and revised both aspects of your comment in the Methods and Material section as the following.

“The **Region of Interest (ROI)** was defined as the extraction socket, extending from the most coronal aspect of the alveolar bone crest to the apical portion of the socket. The ROI was segmented based on grayscale thresholding to differentiate between mineralized and non-mineralized tissues.”

*5.7) Immunohistochemical staining: Why did the authors use only OPG and RANKL markers? In the early stages, there are many other important markers that could have been investigated, such as osteopontin, osteocalcin, BSP, and Runx2.*

Our response: Thank you for this comment. We selected OPG (osteoprotegerin) and RANKL (receptor activator of nuclear factor-kappa B ligand) as our primary markers because they are critical regulators of osteoclast differentiation and activity, which play a central role in the early stages of bone resorption and remodeling. The balance between RANKL and OPG is a key factor in osteoclast-osteoblast coupling, making these markers highly relevant for investigating the early events of osteomucosal healing.

We acknowledge that other markers, such as osteopontin, osteocalcin, bone sialoprotein (BSP), and Runx2, could provide additional insights into bone matrix formation, mineralization, and osteoblast differentiation. However, we did not include these information in this study because we believe bone formation can be objectively assessed by examining calcified new bone structure using uCT and histology. Nonetheless, we acknowledged that these markers are indeed important for understanding later stages of bone healing. To address this limitation, we are planning follow-up studies that will incorporate these markers using immunohistochemical staining and molecular analyses, such as single-cell RNA sequencing (scRNA-seq), to further explore the dynamics of gene and protein expression during osteomucosal healing (Please refer to the answer #4.4).

*5.8) Statistical Analysis: What do the authors mean by "To compare the group differences" if only one group is being analyzed at different time points? Why were so many significance factors used? Since you mentioned that the p-value is < 0.05, the other p-values shown are already within this range.*

Our response: Thank you for pointing out the need for clarification regarding our statistical analysis. We recognize that the phrasing of "group differences" was imprecise. In this study, we did not analyze differences between groups but rather compared changes in measured parameters (e.g., wound closure, collagen deposition, osteoclast activity) across different time points within the same group. The term "group differences" has been revised to reflect comparisons across time points.

Regarding the multiple significance factors, we agree that it was unnecessary to report all p-values below the threshold of <0.05. In the revised manuscript, we have simplified the statistical reporting by clearly stating the threshold for significance (p < 0.05) and removing redundant details about p-values that fall within this range.

*5.9) RESULTS: In general, the results are not well presented, especially in the description. They are confusing, and it is not possible to correlate them with what is being shown in the images, which also lack sufficient resolution for the message the author intends to convey to the readers. I suggest using images with higher magnification.
For example: "By day 3, roughened bone surfaces were observed in the tooth-extracted sockets, suggesting that bone remodeling had started through bone resorption. As a result of this bone remodeling, the sharp edge of the buccal wall margin of the extraction socket became rounded by day 4." This cannot be visualized properly. I suggest using arrows or asterisks to highlight what you would like to show in the images. Once again, the study cited in your references by Vieira is well illustrated in terms of the images.*

Our response: Thank you for your feedback regarding the presentation of our results and the quality of the images. We understand the importance of clear and detailed visualization to effectively communicate our findings. In response, we have made the following revisions:

🡪 Purpose of the Current Magnification: The current magnification was deliberately chosen to capture the entire socket area and provide an overview of the morphological changes occurring during the healing process. We recognize that this broader view may lack the detailed resolution needed for certain features, so we have added additional higher-magnification images of key areas to provide greater clarity.

🡪 Annotation of Figures: To improve the interpretability of the images, we have added arrows, asterisks, and annotations to highlight key features such as roughened bone surfaces (day 3) and rounded buccal wall margins (day 4). This ensures that readers can directly correlate the text with the images.

🡪 Consultation of Vieira et al.: We reviewed the study by Vieira et al. for guidance on presenting bone remodeling processes. While their study focuses on long bones, we adapted their principles to enhance the visualization of our findings in the extraction socket.

🡪 Revised Results Section: The results description has been updated to explicitly reference the annotated images and clarify the key observations.

*5.10) Woven bone formation: You have access to a high-resolution 3D imaging tool, but it is not being used to its full potential. I suggest utilizing better evaluation parameters, as it was not described how the region of interest (ROI) was selected. Once again, I recommend referring to the studies by Vieira and Bouxsein (2010) DOI: 10.1002/jbmr.141 for better guidance.*

Our response: Thank you for your valuable feedback. We agree that the ROI selection and evaluation parameters for woven bone formation should be more clearly described. We have revised the Methods section to explicitly define how the ROI was selected for microCT analysis. Specifically, the ROI was selected as the socket area, encompassing the extraction site from the alveolar crest to the apical portion of the socket. Please refer to answer #5.6 for more details.

Regarding the reference by Vieira and Bouxsein (2010), we agree that this reference provides an excellent protocol for evaluating tooth-extracted area. However, there are subtle but significant difference between our study and theirs in that, our study extracted molars, leading to open wound followed by healing of both soft and hard tissues, while their study extracted incisors and evaluated closed space within the bone. As such, our study requires a novel way of defining ROI, and we have defined in the Materials and Methods Section.

*5.11) Osteoclast formation: I am unable to visualize the osteoclasts present. While we know how the histological sections were made and their positioning, I suggest providing images with higher magnification specifically of the representative areas where these cell types need to be visualized.*

Our response: Thank you for your feedback regarding the visualization of osteoclasts. The revised figures have been updated in the manuscript, and the corresponding figure captions now explicitly describe the location and magnification of the osteoclasts to better correlate with the text.

*5.12) Discussion: The discussion is very bold and poorly supported by the literature. Many statements are not backed by existing references. Additionally, it is confusing, as it moves back and forth on the same topic across several paragraphs. I suggest a thorough rereading and restructuring to improve clarity and coherence.*

Our response: We appreciate this feedback and have thoroughly revised the discussion to ensure statements are well-supported with references. We also restructured the discussion to maintain a coherent flow, grouping related points together and ensuring that each paragraph builds logically on the previous one.

*5.13) "Therefore, these observations indicate that new bone formation in the tooth-extraction socket occurs in two ways: 1) osteoclast-dependent bone formation from the existing bone surface via osteoclast-osteoblast coupling, and 2) osteoclast-independent de novo bone formation via intramembranous ossification." - How can you strongly affirm this if your sample size is low, and you have only evaluated histomorphometric parameters? Such imply may require more robust data, including gene and protein expression analysis, and a larger sample size to provide stronger evidence.*

Our response: Thank you for your feedback. We acknowledge that our sample size (N=6) is not large; however, the data obtained were highly consistent across all samples, providing confidence in the reliability of our findings within the significant level of difference. Additionally, the use of histomorphometric analysis allowed us to capture distinct patterns of bone formation and cellular activity that align with established mechanisms reported in the literature.

That being said, we agree that additional evidence, including molecular and protein expression analyses, would strengthen the conclusions regarding the two pathways of bone formation. We have revised the manuscript to clarify that our results suggest the presence of osteoclast-dependent and osteoclast-independent bone formation mechanisms. Furthermore, we are actively pursuing follow-up studies to validate these findings using larger sample sizes and advanced techniques such as single-cell RNA sequencing (scRNA-seq) and immunohistochemistry for markers like Runx2, osteopontin, and BSP.

To address your concern directly, we have tempered the language in the discussion to better reflect the preliminary nature of these findings, while emphasizing the consistency of our results. Furthermore, we have included clinical implications that can potentially be observed in typical clinical settings including alveolar osteitis, MRONJ, and cicatrization.

*5.14) "The reason for this preferential presence of osteoclasts is presently unclear; however, it seems intuitive that nature has evolved to prevent osteoclasts from targeting the newly forming bone during the healing process to increase the efficiency of new bone formation." - If it is unclear, how can you suggest this information? Based on what evidence or reasoning is this claim being made? It is important to provide solid references or data to support such hypotheses, especially when the mechanism is not yet fully understood.*

Our response: Thank you for pointing out the need to clarify and substantiate this hypothesis. We acknowledge that the mechanism underlying the preferential presence of osteoclasts on pre-existing bone surfaces remains poorly understood. In our discussion, we aimed to present a plausible hypothesis based on the observed spatial distribution of osteoclasts in our study and prior literature suggesting that targeting pre-existing bone surfaces is critical for efficient remodeling and repair. However, we understand the importance of providing evidence to support such claims.

To address your concern, we have revised the manuscript to explicitly identify this as a hypothesis and have moderated the language to avoid over-interpretation. We have also added references to prior studies that report spatially regulated osteoclast activity during bone healing. Additionally, we have highlighted the need for future investigations to elucidate the molecular mechanisms driving this preferential localization.

*5.15) The conclusion is not clear, and the text ends abruptly with a paragraph that seems to suggest continuity, followed by another strong, unreferenced statement: "Unlike other parts of the body, the healing of the soft and hard tissues in the oral cavity often occurs simultaneously after a traumatic event such as tooth extraction." A conclusion needs to be well-defined and should summarize the key findings in a clear and coherent manner, avoiding unsupported or vague statements.*

Our response: We have rewritten the conclusion to clearly summarize the key findings, emphasizing the unique characteristics of osteomucosal healing while avoiding unsupported claims. The revised conclusion highlights the study's contributions and outlines future directions in a coherent and concise manner.
